# Editorial: Dissecting the Intraflagellar Transport System in Physiology and Disease: Cilia-Related and -Unrelated Roles

**DOI:** 10.3389/fcell.2020.615588

**Published:** 2020-11-27

**Authors:** Francesca Finetti, Junmin Pan, Hongmin Qin, Benedicte Delaval

**Affiliations:** ^1^Department of Life Sciences, University of Siena, Siena, Italy; ^2^Ministry of Education Key Laboratory of Protein Sciences, Tsinghua-Peking Center for Life Sciences, School of Life Sciences, Tsinghua University, Beijing, China; ^3^Department of Biology, Texas Agricultural and Mechanical University, College Station, TX, United States; ^4^CRBM, Univ Montpellier, CNRS, Montpellier, France

**Keywords:** intraflagellar transport (IFT), IFT-A and IFT-B complexes, IFT interactors, IFT- related diseases, protein trafficking

A central finding in our understanding of the biology of the primary cilium has been the discovery by Rosembaum's laboratory of a new multimolecular machinery—the Intraflagellar transport (IFT) system (Kozminski et al., 1993). This system is responsible for protein trafficking from the cell body to the ciliary tip and is required for cilia formation and maintenance. Starting from its discovery in 1993 in the green alga *Chlamydomonas reinhardtii*, the IFT system has been extensively studied for many years to characterize the composition and function of the IFT particles in various organisms. IFT particles have been shown to comprise two complexes, namely IFT-A and IFT–B, which move as linear arrays along the axonemal doublet microtubules, transporting specific cargos in the retrograde and anterograde direction through their interaction with molecular motors.

This Research Topic describes different biochemical properties and biological functions of IFT proteins and provides an overall perspective and new insight into the IFT system. Long and Huang focus their attention on mechanisms in ciliary membrane protein trafficking and systematically dissect the different pathways that can be exploited to allow the precise transport and localization of ciliary proteins which are essential not only for ciliogenesis but also for sensory function of cilia. In an Original Research article, Sorusch et al. study the molecular link between the IFT system and the Usher syndrome (USH) protein network, which has been involved in ciliary transport processes. Mutations in USH genes, among which the USH1G gene SANS (scaffold protein containing ankyrin repeats and SAM domain), have been associated with the development of an autosomal recessive disease characterized by vision and hearing loss, accompanied by vestibular dysfunction. Sorusch et al. demonstrate that the USH1G protein SANS, known for its role in protein delivery toward the cilium, directly binds the IFT-B complex proteins IFT52 and IFT57. Interestingly, this report suggests that SANS may be required for the transport of IFT-B molecules to the ciliary base, the assembly of the IFT-B complexes and its delivery into the cilium. Pathologic mutations in the N-terminus of SANS cause the loss of SANS binding to IFT-B molecules probably accounting for the defective ciliary transport processes in photoreceptor cells associated to USH.

Focusing on the vesicular trafficking roles of IFT subunits, Yang and Huang review a body of literature on the role of IFT proteins in ciliated cells but also highlight the contribution of the IFT system to events that are not related to cilium assembly and occur in cells lacking a primary cilium. These include the immunological synapse assembly in the non-ciliated T cells and the vesicular transport targeted to the postsynaptic dendritic terminal in secondary retinal neurons. The relevance of IFT proteins outside the ciliary compartment is also discussed by Vitre et al., who review the associations of IFT proteins with microtubules and motors as well as the non-ciliary roles of IFT proteins in cell division. One novel and timely aspect is discussed by Finetti et al. In this review, the authors focus their attention on the involvement of IFT proteins in degradation pathways in ciliated and non-ciliated cells. In particular, they report direct and indirect roles of the IFT system as a mediator of autophagy by orchestrating, respectively, the trafficking of key autophagy regulators and lysosomal biogenesis. In an Original Research article, Mun et al. investigated the molecular link between autophagy and cilia assembly, two processes that have been previously reported as functionally correlated. Autophagy promotes ciliogenesis trough the degradation of the ciliopathy protein oral-facial-digital syndrome 1 (OFD1) (Tang et al., 2013). In addition, basal autophagy inhibits cilia formation by removing IFT20. Autophagy inhibition thus promotes primary cilia growth (Pampliega et al., 2013). Mun et al. reported that ciliogenesis was positively regulated by autophagy. They demonstrate that the signaling modules composed of phosphatase and tensin homolog (PTEN), Dishevelled2 (Dvl2), and Aurora kinase A (AurKA) could regulate cilia formation through the autophagy regulator p62. Importantly, the relevance of ciliary and non-ciliary IFT protein's function in the etiology of cystic kidney diseases as well as in cancer has been discussed by Vitre et al. and Yang and Huang, respectively.

In summary, this Research Topic provides a comprehensive overview of the IFT system and highlights that IFT proteins are exploited by the cell to regulate not only ciliogenesis but also processes as diverse as signaling pathways, immunological synapse assembly, endocytic and exocytic vesicular traffic, extracellular vesicle release, cytokinesis, and autophagy. This Research Topic also highlights that further investigations based on recent technical development in cell biology are required to assess the ciliary and non-ciliary contribution of IFT proteins in physiological and pathological processes. We hope that the original articles and topical reviews presented in this Research Topic may provide useful insights to inspire future research that unravels the complexity of IFT system.

## Author Contributions

All authors listed have made a substantial, direct and intellectual contribution to the work, and approved it for publication.

## Conflict of Interest

The authors declare that the research was conducted in the absence of any commercial or financial relationships that could be construed as a potential conflict of interest.
